# The First Report of a Missense Variant in *RFX2* Causing Non-Syndromic Tooth Agenesis in a Consanguineous Pakistani Family

**DOI:** 10.3389/fgene.2021.782653

**Published:** 2022-01-25

**Authors:** Sher Alam Khan, Saadullah Khan, Noor Muhammad, Zia Ur Rehman, Muhammad Adnan Khan, Abdul Nasir, Umm-e- Kalsoom, Anwar Kamal Khan, Hassan Khan, Naveed Wasif

**Affiliations:** ^1^ Department of Biotechnology and Genetic Engineering, Kohat University of Science and Technology (KUST), Kohat, Pakistan; ^2^ Dental Material, Institute of Basic Medical Sciences, Khyber Medical University Peshawar, Peshawar, Pakistan; ^3^ Department of Molecular Science and Technology, Ajou University, Suwon, South Korea; ^4^ Department of Biochemistry, Hazara University, Mansehra, Pakistan; ^5^ Institute of Human Genetics, University of Ulm, Ulm, Germany; ^6^ Institute of Human Genetics, University Hospital Schleswig-Holstein, Campus Kiel, Kiel, Germany

**Keywords:** tooth agenesis, non-syndromic, *RFX2*, missense variant, hedgehog and fibroblast growth factor signaling pathways

## Abstract

**Background:** The syndromic and non-syndromic congenital missing teeth phenotype is termed tooth agenesis. Since tooth agenesis is a heterogeneous disorder hence, the patients show diverse absent teeth phenotypes. Thus identifying novel genes involved in the morphogenesis of ectodermal appendages, including teeth, paves the way for establishing signaling pathways.

**Methods and Results:** We have recruited an autosomal recessive non-syndromic tooth agenesis family with two affected members. The exome sequencing technology identified a novel missense sequence variant c.1421T > C; p.(Ile474Thr) in a regulatory factor X (RFX) family member (RFX2, OMIM: 142,765). During the data analysis eight rare variants on various chromosomal locations were identified, but the co-segregation analysis using Sanger sequencing confirmed the segregation of only two variants *RFX2*: c.1421T > C; p.(Ile474Thr), *DOHH*: c.109C > G; p.(Pro37Ala) lying in a common 7.1 MB region of homozygosity on chromosome 19p13.3. Furthermore, the online protein prediction algorithms and protein modeling analysis verified the *RFX2* variant as a damaging genetic alteration and ACMG pathogenicity criteria classified it as likely pathogenic. On the other hand, the *DOHH* variant showed benign outcomes.

**Conclusion:** *RFX2* regulates the Hedgehog and fibroblast growth factor signaling pathways, which are involved in the epithelial and mesenchymal interactions during tooth development. Prior animal model studies have confirmed the expression of *rfx2* at a developmental stage governing mouth formation. Moreover, its regulatory role and close association with ciliary and non-ciliary genes causing various dental malformations makes it a potential candidate gene for tooth agenesis phenotype. Further studies will contribute to exploring the direct role of *RFX2* in human tooth development.

## Introduction

Tooth agenesis (TA) is a craniofacial malformation characterized by the absence of one or more teeth due to failure in the early stages of odontogenesis (Letra et al., 1993). TA is one of the most commonly occurring orofacial congenital anomalies in humans, with a prevalence of ∼3–11% depending upon the ethnicity and geography of the population (Larmour et al., 2005; Shimizu and Maeda, 2009). Population-based studies have shown that the incidence of third molar agenesis (20%) is the most commonly known missing teeth phenotype. Excluding third molars, the prevalence of permanent teeth agenesis is higher (1.6–9.6%) than the primary dentition (0.5–0.9%) (Vastardis, 2000). Based on the number of missing teeth, TA can be classified into three categories: hypodontia (<six missing teeth), oligodontia (>six missing teeth), and anodontia (congenital absence of all primary and permanent teeth) (Stockton et al., 2000; De Coster et al., 2009). Hypodontia is relatively a common condition (1.6–6.9%) as compared to oligodontia (0.14%), while anodontia is extremely rare (Schalk-van der Weide et al., 1992; Polder et al., 2004; Al-Ani et al., 2017). Environmental (infections, trauma, chemotherapy, and radiotherapy) and genetic factors have been reported to cause TA phenotypes, but the latter is the most common cause of TA (Chhabra et al., 2014). Tooth agenesis shows autosomal recessive/dominant (Murakami et al., 2017; Park et al., 2019) and X-linked recessive/dominant patterns of inheritances (Rasool et al., 2008; Sarkar et al., 2014). Formerly, the monogenic inheritance was considered for TA in the previous studies, but recently several studies have proposed oligogenic patterns (Dinckan et al., 2018; Du et al., 2018), supporting the concept of mutational load in human genetic disorders (Posey et al., 2017).

Tooth development is accomplished in a highly coordinated and genetically controlled fashion (Kapadia et al., 2007; Brook, 2009) and is regulated via some molecules involved in the signaling cascades (Kapadia et al., 2007), initiating a series of reciprocal interactions between the epithelium and underlying mesenchyme (Thesleff, 2006). The regular expression of several hundred genes is essential for tooth development (Thesleff, 2014). Genetic alterations in numerous genes have been reported to arrest tooth development in mice and/or humans (Kapadia et al., 2007; Fleischmannova et al., 2008; Brook, 2009; Thesleff, 2014). Four signaling pathways (FGF, BMP, Wnt, and SHH) lead the human tooth formation. In addition, a large number of genes encoding various components of these pathways are either directly or indirectly involved in various tooth conditions (Neubüser et al., 1997; Jussila and Thesleff, 2012). Their loss of function or gain of function may distort the signaling cascades and cause various orofacial anomalies (Nieminen, 2009).

Based on the clinical manifestations, TA can be divided into two sub-categories: congenital non-syndromic tooth agenesis (NSTA) and syndromic tooth agenesis (STA) (Yu et al., 2019). In addition, TA is a prominent feature of more than 300 syndromes (Dinckan et al., 2018), involving primarily in oro-facial cleft and ectodermal dysplasia (Nieminen, 2009), including Witkop syndrome (OMIM: 189,500) (Jumlongras et al., 2001), hypohidrotic ectodermal dysplasia (HED) (Parveen et al., 2019; Khan et al., 2020), Ellis-van Creveld syndrome (EVC, OMIM: 225,500) (Niceta et al., 2018), odonto-onycho-dermal dysplasia (OODD, OMIM: 257,980) (Adaimy et al., 2007) and Bloch-Sulzberger syndrome (OMIM: 308,300) (Si and Liu, 2018).

The genetic etiology of tooth agenesis is heterogeneous. To date, approximately nine genes have been identified to cause different NSTA conditions. Based on clinical phenotypes of TA in the literature and OMIM database (https://www.omim.org/), it can be generalized that some of the genes cause only NSTA, but others are involved in both the NSTA and STA. For instance, *GREM2* (OMIM: 608,832) cause non-syndromic hypodontia (NSH). Similarly, *LRP6* (OMIM: 603,507) cause non-syndromic oligodontia (NSO), while *EDARADD* (OMIM: 606,603) and *AXIN2* (OMIM: 604,025) may cause both the NSO and STA. Four genes including, *MSX1* (OMIM: 142,983), *EDAR* (OMIM: 606,095), *WNT10A* (OMIM: 606,268) and *EDA* (OMIM: 300,451) may cause NSH, NSO and STA, while *PAX9* (OMIM: 167,416) is involved in causing only NSH and NSO (Fournier et al., 2018).

We aimed to characterize the clinical features of an autosomal recessive Pakistani family displaying tooth agenesis and to identify an underlying genetic cause. We present a comprehensive clinical investigation followed by exome sequencing analysis. The exome sequencing data revealed a novel biallelic variant c.1421T > C; p.(Ile474Thr) in 13-exon of *RFX2*.

## Materials and Methods

### Family Recruitment, Pedigree Construction, and Blood Collection

**FIGURE 1:**
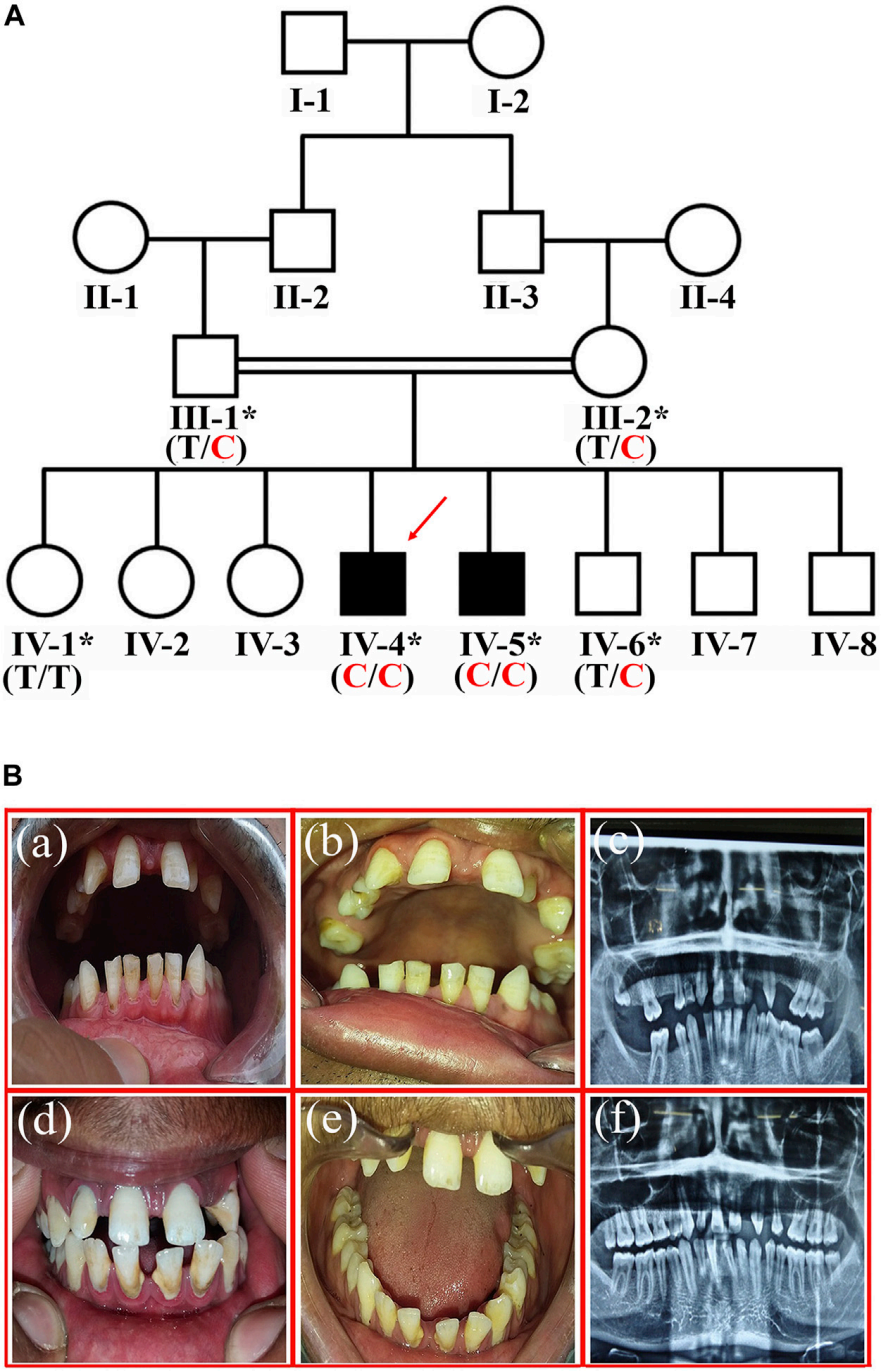
**(A)** Pedigree of the family showing segregation of *RFX2* variant c.1421T > C; p.(Ile474Thr) in an autosomal recessive fashion. The asterisks show participating individuals. The red arrow indicates the index patient subjected for exome sequencing. C shows the disease-allele, while T is the wild-type allele **(B)**. The representation of tooth agenesis. **(a)**, and **(b)** are the clinical features of patient IV-4 showing generalized spacing in the anterior region of maxilla and mandible. **(c)** the Orthopantomogram (OPG) of patient IV-4 shows the absence of permanent maxillary lateral incisors, left maxillary first premolar, and all four 2nd premolars (mandibular and maxillary). It also shows the missing right maxillary second molar, left maxillary third molar, right mandibular second and third molars. **(d)**, and **(e)** are the clinical photographs of patient IV-5, showing the generalized spacing in the anterior region of the maxilla and mandible. **(f)** is the Orthopantomogram (OPG) of patient IV-5, showing the absence of permanent maxillary lateral incisors, maxillary second premolars, and right maxillary first premolar.

A four-generation consanguineous sixteen membered family with two affected individuals (IV-4 and IV-5) with missing teeth phenotype was recruited from the southern region of Khyber Pakhtunkhwa province, Pakistan. Pedigree was constructed based on the information provided by the father (III-1) of the affected members (Figure 1A). Informed written consent was taken from all the participants. The Research and Ethical Committee (REC) of Kohat University of Science and Technology (KUST), Kohat, Pakistan, approved the study protocols, strictly following the recommendations of the Declarations of Helsinki.

### DNA Extraction

Genomic DNA was isolated from the whole peripheral blood of the patients (IV-4 and IV-5) and other family members (III-1, III-2, IV-1 and IV-6) by using the GenElute™ blood genomic DNA kit (Sigma-Aldrich MO, United States). Qubit Fluorometer (ThermoFisher Scientific, United States) was used for the quantification of DNA.

### Exome Sequencing, Alignment and Variant Calling

A 100 ng/μl DNA of two members (IV-1 and IV-4) was used for exome sequencing. The sequencing libraries of the DNA were prepared with the SeqCap EZ human exome library v2.0 kit. The sequencing was done on Illumina HiSeq 4000 sequencing machine via a paired-end 100-bp protocol (Hussain et al., 2013). The filtration of primary data was carried out by the Illumina real-time analysis (RTA) software v1.8. Afterward, the mapping of the reads to the human reference genome build GRCh37/hg19 (http://www.genome.ucsc.edu/) was performed using the BWA-SW alignment algorithm. Picard tools were used to improve the read quality, and Genome Analysis Toolkit (GATK) was used for realignment and base quality score recalibration. The calling of single nucleotide polymorphisms (SNPs) and short insertions/deletions (INDELs) was performed by Platypus, Haplotype Caller, and Mpileup programs and further filtration was carried out through variant quality score calibration (VQSR) using GATK. The ALLEGRO program identified several large runs of homozygosity (ROH) based on multipoint linkage analysis. For the coverage analysis of CNV detection, CNMOPS and ExomeDepth algorithms were utilized. In addition, the COMBINE and FUNC algorithms were used to combine the data and the annotation of functional variants.

### Variant Search, Classification and Sanger Sequencing

The Varbank pipeline v2.26 (https://varbank.ccg.uni-koeln.de/) of Cologne Center for Genomics (CCG), Cologne, Germany, was utilized for the exome data analysis. The mean coverage of the data was 85%, while at 20X and 10X, the coverage of the targeted bases was 93.6 and 96.6%, respectively. A panel of genes, including *MSX1*, *PAX9*, *AXIN2*, *FGFR1*, *IRF6*, *LRP6*, *WNT10A*, *WNT10B*, *EDAR*, and *EDARADD*, underlying various dental malformations, was also filtered out to exclude the involvement of any copy number variations, missense, nonsense, or compound heterozygous variants in these genes.

Considering the consanguinity among the parents, ROH in the affected members (≥5 Mb) were identified. Later, a variant search was carried out in the ROH to find out rare homozygous variants. Furthermore, an exome-wide search irrespective of ROH was also executed to search for rare biallelic variants. Based on the autosomal recessive inheritance pattern, allele read frequency (75%-100%) for homozygous changes and allele frequency (<1%) for recessive variants was used. VarSome (Kopanos et al., 2019), Human Gene Mutation Database (HGMD) Professional 2019.4, and Database of Single Nucleotide Polymorphisms (dbSNP) were utilized for the evaluation of variants. Genome Aggregation Database v.2.1.1 (gnomAD; https://gnomad.broadinstitute.org/) was consulted to establish the minor allele frequency (MAF; value < 0.01) of the variants. An in-house database of 511 exomes of patients with diverse phenotype and another dataset of 21-exomes of ethically matched Pakhtun patients along with 90-exomes of other Pakistani patients of various ethnic backgrounds were employed as a control. ACMG guidelines were consulted for the classification of pathogenic, likely pathogenic, uncertain significance and benign variants (Richards et al., 2015).

In addition, a thorough search was performed on the variant data of chromosome X, considering the gender of the affected members in the pedigree diagram, but no bonafide variant, neither in *EDA* nor in other X-linked genes, was identified.

After performing the exome data analysis, all the gene variants were filtered and validated in various databases including dbSNP (https://www.ncbi.nlm.nih.gov/snp/), 1000 genome project (https://www.internationalgenome.org/), EVS (https://evs.gs.washington.edu/EVS/), and gnomAD (https://gnomad.broadinstitute.org/).

The reference sequences were obtained from the University of California Santa Cruz (UCSC) genome database browser (http://genome.ucsc.edu/cgi-bin/hgGateway). Amplifx version 2.1 (https://inp.univ-amu.fr/en/amplifx-manage-test-and-design-your-primers-for-pcr) was used for designing the primers for the amplification of the regions of interest. First, a genomic sequence of 700 bp up-and-downstream from the position of the rare variant was scanned to design an appropriate primer pair. Then, the PCR amplification of the regions of interest was carried out, and the Exo-Sap protocol (https://www.thermofisher.com) was used for purifying the PCR products. Next, the DNA sequencing was performed on the ABI3730 genetic analyzer with BigDye chemistry v3.1. For aligning the sequences against the reference sequence, a sequence alignment tool, BioEdit version 6.0.7 (http://www.mbio.ncsu.edu/BioEdit/bioedit.htm) was utilized.

### Pathogenicity Prediction and Protein Sequence Alignment

*In silico* analysis was performed using different pathogenicity prediction tools including, 1) Mutation Taster (http://www.mutationtaster.org/), 2) PolyPhen-2 (http://genetics.bwh.harvard.edu/pph2/), 3) PROVEAN (http://provean.jcvi.org/seq_submit.php), 4) PhD-SNP (https://snps.biofold.org/phd-snp/phd-snp.html), 5) I-Mutant2.0 (https://folding.biofold.org/i-mutant/i-mutant2.0.html), 6) SIFT (https://sift.bii.a-star.edu.sg/), 7) MutPred2 (http://mutpred.mutdb.org/), 8) FATHMM (http://fathmm.biocompute.org.uk/inherited.html) 9) VarSome (https://varsome.com/) (10) CADD (https://cadd.gs.washington.edu/snv) and 11) GERP (https://bio.tools/gerp).

Clustal Omega tool (https://www.ebi.ac.uk/Tools/msa/clustalo/) was used for the multiple sequence alignment of RFX2 proteins among different species including human (ENST00000303657.10), Chimpanzee (ENSPTRT00000080337.1), Gorilla (ENSGGOT00000060572.1), Gibbon (ENSNLET00000048810.1), Guinea (ENSCPOT00000022084.2), Opossum (ENSMODT00000058801.1), Koala (ENSPCIT00000013029.2), Cat (ENSFCAT00000038434.3), Panda (ENSAMET00000008566.1), Donkey (ENSEAST00005036789.1) and Dog (ENSCAFT00000029731.4).

### Protein Structure Prediction

The homology modeling technique was utilized to construct the three-dimensional structures of RFX2 and DOHH proteins using I-TASSER (Iterative Threading ASSEmbly Refinement) server (Yang et al., 2015). For wild-type and mutant (p.Ile474Thr) RFX2 modelling, we used PDB (Protein Data Bank) ID: IDP7 (intrinsically disordered protein-7) structure as a template with an 84% structure homology. In the case of DOHH-p.Pro37Ala, we used the crystal structure of human DOHH (PDB ID: 4D50). A similar energy minimization strategy was utilized for the wild-type and mutant structures. Finally, structures were visualized with PyMOL software (www.pymol.org) (Delano, 2002).

## Results

### Clinical Report

The affected individuals (IV-4, 29-years, and IV-5, 24-years) showing variable missing teeth conditions were recruited at the dentistry department, Khyber Medical University, Peshawar, Khyber Pakhtunkhwa (KP), Pakistan. A dental specialist performed a clinical evaluation of the patients. The unaffected members (III-1, III-2, IV-1, IV-6) available for the study (Figure 1A) were also evaluated at the same clinic. The patients showed spacing in the upper jaw between the two central incisors. In addition, intra-oral examination showed the generalized spacing in the anterior region of the maxilla and mandible. During diagnosis, the missing third molars were excluded. In the case of patient-IV-4, a total of nine permanent teeth were missing (maxillary lateral incisors, left maxillary first premolar, all 4 s premolars, the right maxillary second molar, and right mandibular second molar) (Figure 1B a, b). However, in patient-IV-5, five permanent teeth including, maxillary lateral incisors, maxillary second premolars, and right maxillary first premolar, were missing (Figure 1B d, e).

Maxillary central incisors were proclined labially in both patients with patchy chalky white discoloration on the labial surface and around the root of the deciduous first molar. All maxillary teeth, except the central incisors, were small in size, and there was a lower arch spacing due to missing mandibular molars on both sides. Deep attrition in the case of maxillary incisors, canines, and premolars with enamel loss from their incisal surfaces and a taurodontism in the left mandibular third molar was observed. In addition, generalized horizontal bone loss and deep biting were also discerned. Furthermore, there was a clinical midline diastema due to the missing lateral incisors and a thin enamel covering over the dentine, especially in the lower incisor and lower premolars. Periapical radiolucency around the root of the deciduous first molar was also reported (Figure 1B c, f). The unaffected members showed normal permanent dentition in both arches (maxillary and mandibular).

The patients were of standard heights (IV-4 = 152.4 cm, IV-5 = 153.6 cm), and they did not show any neurological, skeletal, and visceral organ defects upon medical examinations. Likewise, the abnormalities of other ectodermal appendages, including hypotrichosis, hyponychia/anonychia, cleft lip/palate, hypo/hyperpigmentation, and hypohidrosis, were ruled out by the medical consultants during the physical investigations of these patients. In addition, the patients did not record any complaints of muscular weakness or any progressive physical lethargies.

### Variant Identification and Co-segregation Analysis

During the exome analysis, several ROH, including a 42.7 MB ROH on chromosome 1p31.1–12, a 19.8 MB ROH on chromosome 7q31.32-q34, a 13.8 MB ROH on chromosome 6p21.32-q23.3, and a 7.1 MB ROH on chromosome 19p13.3–13.2, were identified. These chromosomal locations carried eight rare homozygous variants, including c.1891C > T; p.(Leu631Phe) (NM_003,594.4) in *TTF2* (1p13.1), c.980C > T; p.(Thr327Met) (NM_207,163.3) in *LMOD2* (7q31.32), c.3732G > T; p.(Leu1244Phe) (NM_173,569.4) in *UBN2* (7q34), c.406C > A; p.(Pro136Thr) (NM_053,278.2) in *TAAR8* (6q23.2), c.122T > G; p.(Phe41Cys) (NM_002,123.4) in *HLA-DQB1* (6p21.32), c.266T > C; p.(Ile89Thr) (NM_152,990.3) in *PXT1* (6p21.31), c.1421T > C; p.(Ile474Thr) (NM_000,635.4) in *RFX2* (19p13.3), and c.109C > G; p.(Pro37Ala) (NM_031,304.4) in *DOHH* (19p13.3) (Table 1). The exome-wide search regardless of ROH identified ∼120 homozygous variants on various chromosomal locations. Upon applying the variant validation criteria described in Materials and Methods, we could not find any rare variant other than the variants mentioned in Table 1. Therefore, these variants were considered for the co-segregation analysis.

**TABLE 1:** Rare coding variants prioritized from exome sequencing data, which were tested for segregation.

HGNC	Chr	c.DNA Change	Amino acid change	Chr. Position	Ref_ allele	Alt_ allele	Change	EnsProt	Gene ID_ OMIM	RefSeq	Exon	gnomAD v2. 1. 1 Hom/Het/Allele Count	MAF (gnomAD v2. 1. 1)	dbSNP rsID	Segregation
*TTF2*	1	c.1891C > T	p.(Leu631Phe)	117,624,557	C	T	Missense	ENSP00000358478	604,718	NM_003,594.4	10	NR	NR	NR	No
*LMOD2*	7	c.980C > T	p.(Thr327Met)	123,302,620	C	T	Missense	ENSP00000411932	608,006	NM_207,163.3	2	1/28/280,098	0.00009997	rs535251089	No
*UBN2*	7	c.3732G > T	p.(Leu1244Phe)	138,978,040	G	T	Missense	ENSP00000418648	613,841	NM_173,569.4	16	NR	NR	NR	No
*TAAR8*	6	c.406C > A	p.(Pro136Thr)	132,874,237	C	A	Missense	ENSP00000275200	606,927	NM_053,278.2	1	NR	NR	NR	No
*HLA-DQB1*	6	c.122T > G	p.(Phe41Cys)	32,632,832	A	C	Missense	ENSP00000407332	604,305	NM_002,123.4	3	NR	NR	rs9274407	No
*PXT1*	6	c.266T > C	p.(Ile89Thr)	36,368,265	A	G	Missense	ENSP00000419944	NI	NM_152,990.3	4	1/43/282,872	0.0001520	rs767510507	No
*RFX2*	19	c.1421T > C	p.(Ile474Thr)	6,004,291	A	G	Missense	ENSP00000306335	142,765	NM_000,635.4	13	0/32/251,482	0.0001272	rs769861701	Yes
*DOHH*	19	c.109C > G	p.(Pro37Ala)	3,496,704	G	C	Missense	ENSP00000250937	611,262	NM_031,304.4	2	0/2/248,922	0.000008035	rs766767851	Yes

Chr: Chromosome, Ref: reference, Alt: Altered, EnsProt: Ensembl Protein, RefSeq: Reference Sequence g: Genomic, c: Coding, NI: no information, NR: not reported, MAF: minor allele frequency, SNP: single nucleotide polymorphism, SNV: single nucleotide variant, HOM: homozygous, Het: Heterozygous, Allele Count: Total number of alleles.

**FIGURE 2:**
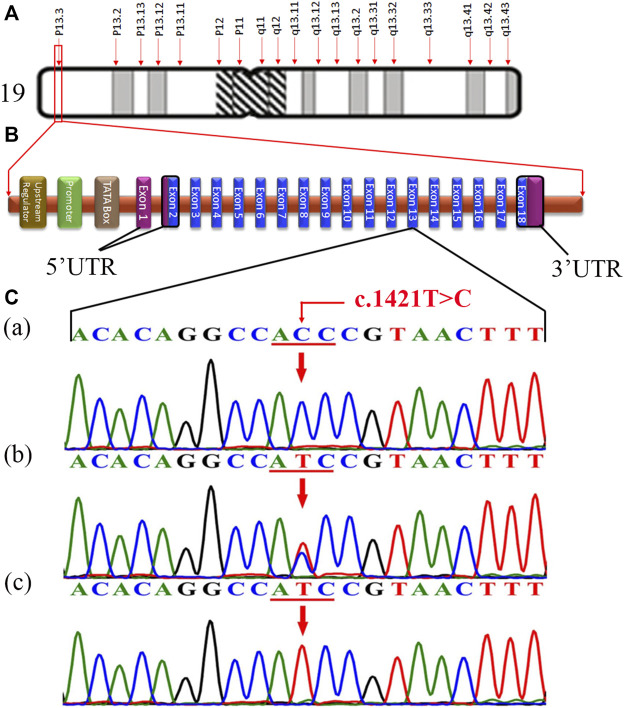
**(A)** Structure of chromosome 19, showing the *RFX2* locus (p13.3) (http://atlasgeneticsoncology.org/ISCN09/Chrom19ISCN09.html). **(B)** Typical structure of *RFX2* containing 18 exons, showing the position of genetic alteration c.1421T > C in 13-exon in the present study (red) (generated manually using the information from ENSEMBL Genome Browser). **(C)** Chromatogram of an unaffected individual (IV-1) in the upper panel **(a)**, a carrier/heterozygous parent (III-1) in the middle panel **(b)**, and a homozygous affected individual (IV-4) in the lower panel **(c)**.

The fine-mapped variants were sequenced via Sanger sequencing for the validation and confirmation of segregation. During the co-segregation analysis, the variants in *RFX2* and *DOHH*, located in a common ROH of 7.1 MB on chromosome 19 segregated in the family (Figures 1A, 2C). The *DOHH* variant c.109C > G; p.(Pro37Ala) was predicted benign and tolerated by the online prediction tools, while the variant c.1421T > C; p.(Ile474Thr) in *RFX2* was predicted damaging. Moreover, the *RFX2* variant achieved highly significant CADD (27.3) and GERP (5.1399) scores (Table 2). The ACMG variant classification criteria classified the *RFX2* variant as likely pathogenic, while the *DOHH* variant was benign (Table 2). Hence, the *RFX2* variant is the most likely candidate causing non-syndromic tooth agenesis. An allele frequency (AF) (0.0001272) of this variant has been calculated in gnomAD. This variant is 32 times monoallelically identified in South Asian (17), European (non-Finnish, 13), Latino/Admixed Americans (1), and in a minor population (1), where the total number of alleles is 251,482 while the number of homozygous alleles is zero. From the South Asian population, 30,616 alleles are included with an AF of 0.0005553 in this database. A dbSNP ID rs769861701 has been assigned to this variant. According to our knowledge, this is the first report of a biallelic variation at c.1421T > C in *RFX2*, which is likely to cause human absent teeth phenotype (Figures 2A–C).

**TABLE 2:** *In silico* analysis of the coding variants with scores by using the various online pathogenicity prediction tools.

Gene	Variant	Mutation taster	PolyPhen-2	PROVEAN	PhD-SNP	I-Mutant2.0	SIFT	MutPred	FATHMM	FATHMM-XF	VarSome	CADD PHRED	GERP (NR)	GERP (RS)	ACMG 2015
*TTF2*	c.1891C > T; p.(Leu631Phe)	Disease causing P: 0.99981	Benign Score: 0.075	Neutral Score: 1.428	Disease causing Score: 4	Stability decrease RI Score: 6	Damaging Score: 0.01	Benign Score:0.453	Damaging Score: 2.48	Neutral Score: 0.2507	Likely Benign	22.4	5.25	3.2	NA
*LMOD2*	c.980C > T; p.(Thr327Met)	Disease causing P: 0.99984	Probably Damaging Score: 1.000	Deleterious Score: 5.167	Disease causing Score: 6	Stability decrease RI Score: 4	Damaging Score: 0.01	NS	Damaging Score: 3.09	Damaging Score: 0.7156	Uncertain Significance (Likely Pathogenic)	25.4	5.36	5.36	NA
*UBN2*	c.3732G > T; p.(Leu1244Phe)	Disease causing P: 0.99970	Probably Damaging Score: 0.999	Neutral Score: 0.100	Neutral Score: 7	Stability decrease RI Score: 5	Tolerated Score: 0.22	Benign Score:0.287	Tolerated Score: 1.26	Neutral Score: 0.2586	Uncertain Significance (Likely Benign)	24.2	6.0599	5.17	NA
*TAAR8*	c.406C > A; p.(Pro136Thr)	Disease causing P: 0.99986	Probably Damaging Score: 0.999	Deleterious Score: 7.910	Disease causing Score: 5	Stability decrease RI Score: 8	Damaging Score: 0.00	Pathogenic Score: 0.907	Tolerated Score: 0.11	Damaging Score: 0.8111	Uncertain Significance (Likely Pathogenic)	23.7	4.6199	4.6199	NA
*HLA-DQB1*	c.122T > G; p.(Phe41Cys)	Polymorphism P: 0.9999	Possibly Damaging Score: 0.840	Neutral Score: 2.313	Disease causing Score: 7	Stability decrease RI Score: 7	Tolerated Score: 0.18	Benign Score: 0.513	Tolerated Score: 3.44	Neutral Score: 0.2509	Uncertain Significance (Benign)	0.503	4.3	−8.25	NA
*PXT1*	c.266T > C; p.(Ile89Thr)	Polymorphism P: 0.949	Possibly Damaging Score: 0.848	Deleterious Score: 4.567	Neutral Score: 8	Stability decrease RI Score: 8	Tolerated Score: 0.073	NS	NS	Neutral Score: 0.1218	Uncertain Significance (Benign)	8.011	5.2199	4.07	NA
*RFX2*	c.1421T > C; p.(Ile474Thr)	Disease causing P: 1	Probably Damaging Score: 0.993	Deleterious Score: 4.799	Disease causing Score: 8	Stability decrease RI Score: 9	Damaging Score: 0.01	Pathogenic Score: 0.872	Tolerated Score: 2.95	Damaging Score: 0.8879	Uncertain Significance (Likely Pathogenic)	27.3	5.1399	5.1399	Likely pathogenic (PP1, PP3, PP4, PM1, PM2)
*DOHH*	c.109C > G; p.(Pro37Ala)	Polymorphism P: 0.7041	Benign Score: 0.000	Neutral Score: 1.307	Neutral Score: 7	Stability decrease RI Score: 9	Tolerated Score: 0.73	Benign Score: 0.163	Tolerated Score: 0.58	Neutral Score: 0.09145	Uncertain Significance (Benign)	11.26	4.28	3.24	Benign (PP1, PM2, BP4, BP6)

P: probability, RI: Reliability Index, NS: no score available, NA: not applicable.

### Protein Structure Prediction

**FIGURE 3:**
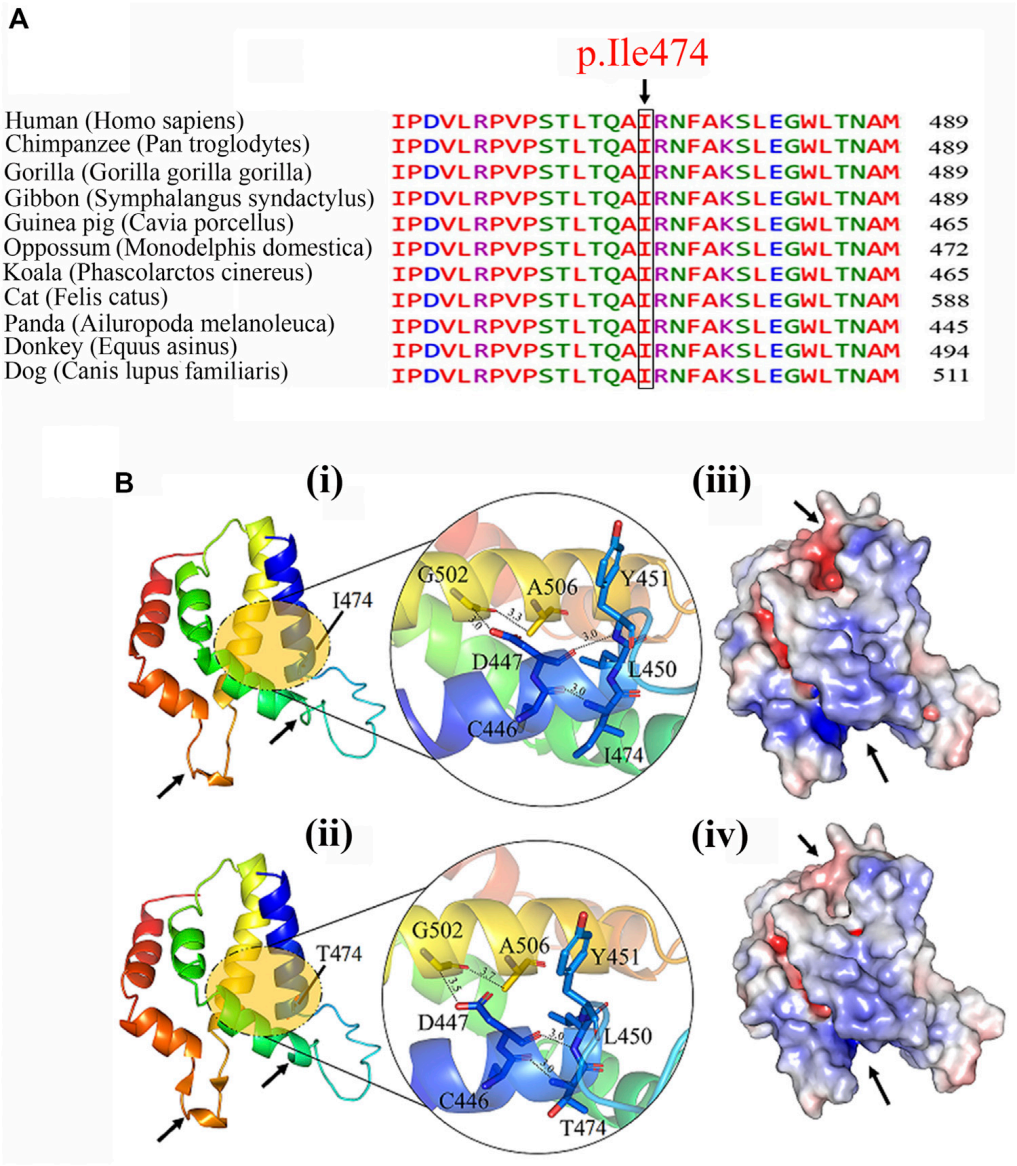
**(A)** Multiple sequence alignment of Ile474 with its mammalian orthologs, showing conserved wild-type residue (Isoleucine) at this position. **(B)** The predicted structure of wild **(i)** and mutant **(ii)** of RFX2 protein. Zoom-up view of interaction pattern of the nearby residues of wild-type and mutant protein. Possible structural effects of substituting conserved isoleucine into a threonine at residue 474 are highlighted with an arrow and surface charge distribution of **(iii)** wild-type and **(iv)** mutant- RFX2.

Multiple sequence alignment revealed conservation of the RFX2-Isoleucine-474 residue, as shown in Figure 3A, and the conserved residue is essential for protein structure determination, stability, and functionality. The structural relevance of the highly conserved Isoleucine residue was determined using the homology modeling technique. We examined and studied both wild-type and mutant structures to discover the structural differences, depicted in Figure 3B i and ii. As illustrated in the zoomed-up view, we detected differences in intramolecular distance among residues in the immediate mutation vicinity. Because of the variation in bonding, there is a local difference in the structure, as indicated by the arrows. We also noticed a difference in surface charge distribution between the two forms of the RFX2 protein, as indicated by the arrows (Figure 3B iii and iv).

**FIGURE 4:**
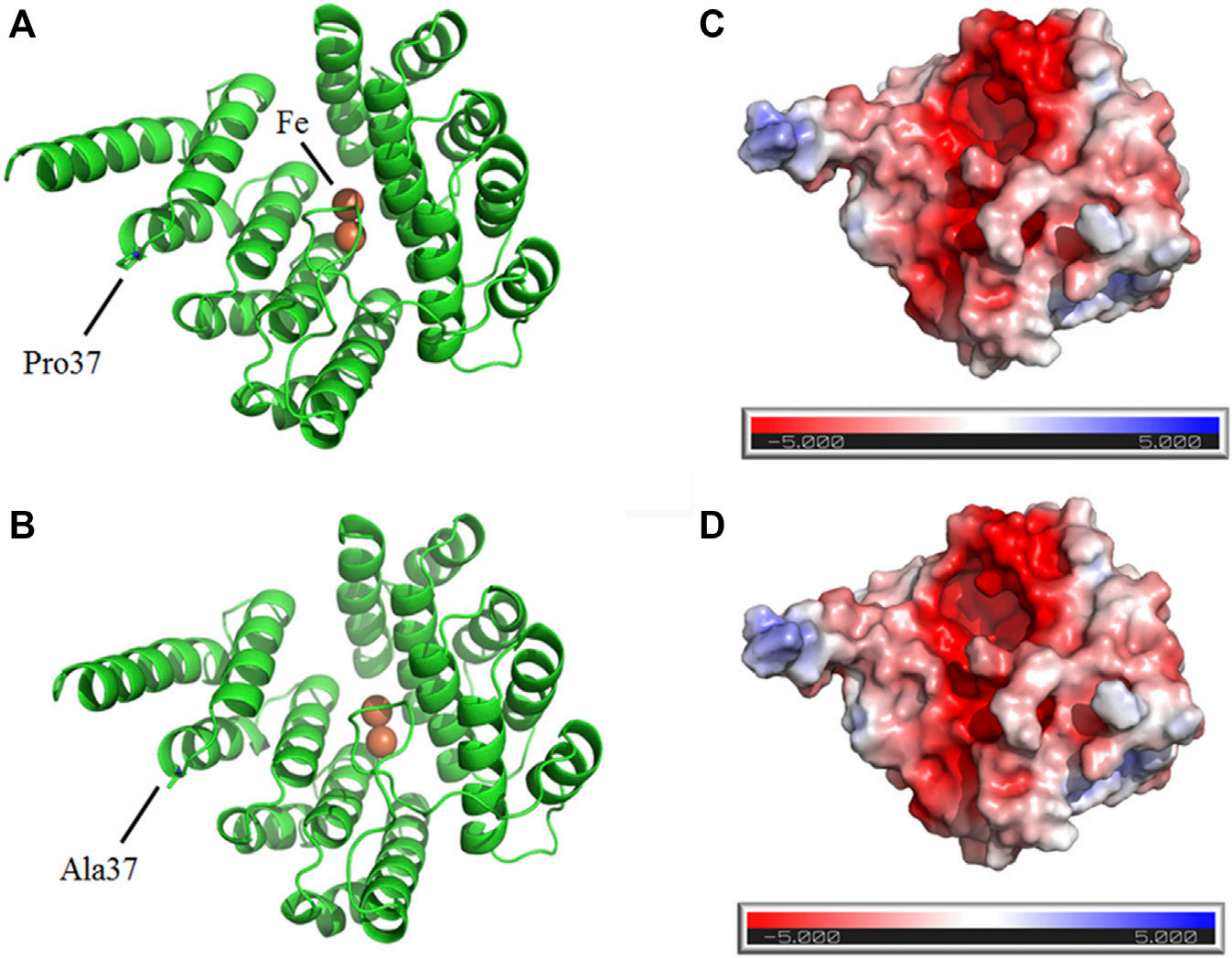
Predicted structures of wild **(A)** and mutant **(B)** DOHH proteins show the surface location Pro37 and Ala37, respectively. The electrostatic analysis of wild-type **(C)** and mutant **(D)** DOHH protein.

On the other hand, Pro37 and Ala37 are located on the surface of wild and mutant DOHH proteins, respectively, suggesting the *DOHH* variant is substantially insignificant (Figures 4A,B). Furthermore, electrostatic analysis of wild-type and mutant DOHH showed that the residue is not present at the interface of DOHH dimeric form and is not involved in any inter-protein and intra-protein interactions (Figures 4C,D).

## Discussion

In this study, we have investigated the genetic cause of the NSTA in a Pakistani family through exome and Sanger sequencing. The affected individuals manifested a generalized spacing in the upper jaw between the two central incisors in addition to congenitally missing permanent maxillary lateral incisors, left and right maxillary first premolars, and mandibular and maxillary second premolars. Besides, the exome data revealed a novel homozygous sequence variant c.1421T > C; p.(Ile474Thr) in *RFX2*, which lies in an extended dimerization domain C of RFX2 protein. According to the ACMG guidelines (2015), this variant has been categorized as a likely pathogenic variant (Table 2).

*RFX2* comprises 18 exons (Figures 2A,B), encoding a 723 amino acids protein (RFX2), and has two isoforms, P48378 and P48378-2 (https://www.uniprot.org/uniprot/). In humans, eight RFX genes (*RFX1*-*RFX8*) have been characterized (Aftab et al., 2008; Sugiaman-Trapman et al., 2018). RFX genes encode transcription factors (TFs) termed RFX transcription factors (RFX-TFs), performing multiple functions in different organisms and have been argued to be involved in the regulation of various cellular and developmental mechanisms (Zaim et al., 2005; Garg et al., 2015). As the RFX genes have essential activities in development hence, the alterations in their sequence may lead to severe pathogenic consequences (Senti and Swoboda, 2008; Choksi et al., 2014). According to Human Gene Mutation Database (HGMD) Professional 2021.2, five missense variants associated with autism (Sanders et al., 2012; Kosmicki et al., 2017; Satterstrom et al., 2020), cerebral palsy (McMichael et al., 2015), and congenital heart defect (Jin et al., 2017) have already been reported in *RFX2*. Here we report a novel sequence variant c.1421T > C; p.(Ile474Thr) in this gene in an autosomal recessive family exhibiting tooth agenesis. As predicted by the bioinformatics tools, this missense variant results in structural deviations in RFX2 protein, leading to significant perturbations or abolishing its function.

*rfx2* expresses in various ciliated tissues in Xenopus, including the neural tube, which leads to the development of the brain, spinal cord and epidermis, gastrocoel roof plate, inner ear, and renal tissues (Chung et al., 2014). In addition, severe cilia-defective embryonic phenotypes were observed in *rfx2* ^-/-^ Xenopus (Chung et al., 2014). Rfx-TFs are involved in the hearing mechanism of mice (Elkon et al., 2015). *rfx2* is highly expressed in testes (Reith et al., 1994), and Wu et al. (2016) have studied the defective spermatogenesis and severe growth retardation in *rfx2* ^-/-^ mice (Wu et al., 2016). In a mouse model study, Bisgrove et al. (2012) have observed the expression of *rfx2* at the late headfold stage (Bisgrove et al., 2012). The headfold stage governs the formation of the heart (Varner et al., 2010) and an oral membrane in the jawed vertebrates, which leads to mouth morphogenesis, including the development of jaws and teeth (Soukup et al., 2013). Considering the role of *RFX2* as a transcriptional factor and its significant expression in various tissues, we hypothesize that it regulates the morphogenesis of multiple tissues and organs in animals and humans, including the formation of craniofacial tissues. Besides, the sequence alterations of *RFX2* may cause various intellectual disabilities, heart anomalies, renal defects, otologic disorders, and abnormal growth of ectodermal appendages, including teeth.

RFX proteins play a central role in the morphogenesis of cilia on the surface of polarized cells (Senti and Swoboda, 2008; Senti et al., 2009). In humans and animals, the primary cilia are involved in dental development, located in the epithelial and mesenchymal tissues during initial stages, differentiation, and formation of complex tissues during tooth development (Kero et al., 2014; Hampl et al., 2017). As the primary cilia are the important signaling centers during vertebrate development (Goetz and Anderson, 2010), any genetic or experimental alteration in their structure or function may lead to defective odontogenesis (Liu et al., 2014), resulting in a change in the tooth formula, size, morphology, position (Hardcastle et al., 1998; Lan et al., 2014), and odontoblast/ameloblast differentiation (Bei, 2009). Moreover, several ciliopathies, including Bardet-Biedl syndrome (BBS), Ellis-van Creveld syndrome (EVC; OMIM: 225,500), Weyers acrofacial dysostosis (WAD; OMIM: 183,530), have been reported in the literature displaying syndromic genetic anomalies, including the dental defects, hence, demonstrating the principal role of primary cilia in tooth morphogenesis (Hampl et al., 2017). Recently, it has been studied that *Rfx2* regulates the expression of genes encoding axonemal dynein subunits, proteins involved in epithelial-mesenchymal transition, and the BBSome elements (Chung et al., 2014). These genes have been reported in human ciliopathies (BBS, EVC, WAD) (Sharma et al., 2008; Reiter et al., 2012). Furthermore, *Rfx2* directly influences the downstream expression of 911 genes, and 180 genes out of those are only the ciliary genes (Chung et al., 2014).

RFX2 is essential for the expression of numerous genes, regulating the Hedgehog (Hh) signaling (Chung et al., 2012; Chung et al., 2014). Sonic hedgehog (*Shh*) is expressed in the dental epithelium and regulates the formation of enamel, dentin, cementum, and soft tissues (Hosoya et al., 2020). A recent study by Neugebauer et al. (2009) revealed that the disruption of FGF signaling through *fgfr1* reduces the expression of *ift88* and two ciliogenic transcription factors *foxj1* and *rfx2* (Neugebauer et al., 2009). An *ift88* null mice exhibit skeletal defects and hypoplastic maxilla, mandible, and supernumerary teeth (Zhang et al., 2003; Ohazama et al., 2009). The pathogenic genetic changes in *fgfr1* also cause phenotypes consistent with those seen in *ift88*. The pathogenic variants in human *FGFR1* have been reported to cause various tooth malformations (Dodé et al., 2003; Pitteloud et al., 2006). In human ciliated cells, *IFT88* (OMIM: 600,595) co-localizes with *OFD1* (OMIM: 300,170), and genetic alterations in *OFD1* cause Orofaciodigital Syndrome-1 (Singla et al., 2010). As the expression of the genes discussed above is dependent on each other and they govern the same signaling pathway, we firmly believe that *IFT88*, *FOXJ1* (OMIM: 602,291), and *RFX2* are the strong candidate genes associated with human development and the genetic variations in these genes may lead to skeletal, oral and maxillofacial anomalies.

## Conclusion

In this study, we report a clinical and molecular diagnosis of a consanguineous NSTA Pakistani family. Exome sequencing and Sanger sequencing identified a novel homozygous missense variant c.1421T > C; p.(Ile474Thr) in a novel candidate gene *RFX2* causing NSTA in this family. We claim that *RFX2*, based on its critical regulatory role in ciliary development, SHH, and FGF signaling, is an essential molecular player in human odontogenesis.

## Data Availability

The datasets presented in this study can be found in online repositories. The names of the repository/repositories and accession number(s) can be found below: ClinVar, SCV001934213.1.
